# Metabolic Impact of Anti-Angiogenic Agents on U87 Glioma Cells

**DOI:** 10.1371/journal.pone.0099198

**Published:** 2014-06-12

**Authors:** Tanja Mesti, Philippe Savarin, Mohamed N. Triba, Laurence Le Moyec, Janja Ocvirk, Claire Banissi, Antoine F. Carpentier

**Affiliations:** 1 Laboratoire de Recherches Biochirurgicales, Université Paris Descartes, Hôpital Européen Georges Pompidou, Paris, France; 2 Chemistry, Structure and Properties of Biomaterials and Therapeutic Agents, Unité Mixte de Recherche 7244, Centre National de la Recherche Scientifique, Université Paris 13 Sorbonne Paris Cité, Bobigny, France; 3 Unité de Biologie Intégrative des Adaptations à l'Exercice, Unité 902, Institut National de la Santé et de la Recherche Médicale, Université d'Evry, Evry, France; 4 Division of Medical Oncology, Institute of Oncology Ljubljana, Ljubljana, Slovenia; 5 Unité de Formation et de Recherche de Santé, Médecine et Biologie Humaine, Université Paris 13, Bobigny, France; 6 Hôpital Avicenne, Assistance Publique-Hôpitaux de Paris, Bobigny, France; University of Nebraska Medical Center, United States of America

## Abstract

**Background:**

Glioma cells not only secrete high levels of vascular endothelial growth factor (VEGF) but also express VEGF receptors (VEGFR), supporting the existence of an autocrine loop. The direct impact on glioma cells metabolism of drugs targeting the VEGF pathway, such as Bevacizumab (Bev) or VEGFR Tyrosine Kinase Inhibitor (TKI), is poorly known.

**Material and Methods:**

U87 cells were treated with Bev or SU1498, a selective VEGFR2 TKI. VEGFR expression was checked with FACS flow cytometry and Quantitative Real-Time PCR. VEGF secretion into the medium was assessed with an ELISA kit. Metabolomic studies on cells were performed using High Resolution Magic Angle Spinning Spectroscopy (HR-MAS).

**Results:**

U87 cells secreted VEGF and expressed low level of VEGFR2, but no detectable VEGFR1. Exposure to SU1498, but not Bev, significantly impacted cell proliferation and apoptosis. Metabolomic studies with HR MAS showed that Bev had no significant effect on cell metabolism, while SU1498 induced a marked increase in lipids and a decrease in glycerophosphocholine. Accordingly, accumulation of lipid droplets was seen in the cytoplasm of SU1498-treated U87 cells.

**Conclusion:**

Although both drugs target the VEGF pathway, only SU1498 showed a clear impact on cell proliferation, cell morphology and metabolism. Bevacizumab is thus less likely to modify glioma cells phenotype due to a direct therapeutic pressure on the VEGF autocrine loop. In patients treated with VEGFR TKI, monitoring lipids with magnetic resonance spectroscopic (MRS) might be a valuable marker to assess drug cytotoxicity.

## Introduction

Glioblastomas (GBMs) are rapidly growing tumors that extensively invade the brain. Despite surgical resection followed by radiation therapy and concomitant temozolomide, the prognostic remains dismal with a median survival of less than 15 months [1]. GBMs secrete high levels of vascular endothelial growth factor (VEGF) that promotes endothelial cell proliferation, blood brain barrier (BBB) permeability, and angiogenesis [2]. VEGF Receptor 1 (VEGFR1) and VEGFR2 are expressed by the vascular endothelial cells. VEGFR2 mediates almost all of the known cellular responses to VEGF [3]. Interestingly, several studies reported that glioma cells not only secrete high levels of VEGF but also express VEGF receptors, supporting the existence of an autocrine loop [4]–[7].

Several anti-angiogenic agents have been developed in the recent years, either targeting the tyrosine kinase of the VEGF receptors or the VEGF itself. Bevacizumab, a monoclonal antibody targeting VEGF, demonstrated a high rate of radiological responses and an increased in progression-free survival in both recurrent [8]–[10] and newly diagnosed GBMs [11]. Cediranib, a VEGFR tyrosine kinase inhibitor, has also been evaluated in GBM patients. However, despite a high level of radiological responses in magnetic resonance imaging (MRI), Cediranib failed to increase progression-free survival and overall survival in a randomized trial [12], [13].

While antiangiogenic treatments produce dramatic reduction of contrast enhancement in MRI, largely due to a reduced BBB permeability, the degree to which these radiological responses are associated with a real tumoricidal effect remains unclear [14]. The inability of routine contrast-enhanced MR imaging to differentiate between a steroid-like effect and cytotoxicity on tumor cells has led to increased interest in magnetic resonance spectroscopy (MRS) to study the metabolic status of tumors in GBM patients [15].

To study the potential cytotoxicity of antiangiogenic agents on gliomas cells themselves, we compared two antiangiogenic agents targeting the VEGF pathway *in vitro*. Gliomas cells were treated with either Bevacizumab or SU1498, a selective VEGFR2 inhibitor [16]. We first assessed the drug effects on cell proliferation, cell morphology and VEGF secretion. We then investigated *in vitro* by High Resolution Magic Angle Spinning Spectroscopy (HR-MAS), the metabolic impact of these treatments on tumor cells. HR-MAS is a very sensitive method for analyzing biological tissue samples that can advantageously be used to determine whether two drugs display or not a similar effect on the cell metabolism [17]–[21]. In addition, HR-MAS can provide useful information on the relevant tumor metabolites to be monitored in patients.

We here report that Bevacizumab minimally affected glioma cells phenotype and metabolism. On the contrary, SU1498 induced a marked increase in lipids and a decrease in glycerophosphocholine. Studying these metabolites by MRS in patients could provide an early surrogate marker of cytotoxicity on tumor cells, and might thus have a significant impact on clinical practice.

## Materials and Methods

### 1. Cell culture and drugs

The U87 cell line (ATCC, Rockville, USA) was maintained in Eagle's minimal essential medium (EMEM) with 10% fetal calf serum, 2 mM L-glutamine, 100 U/mL Penicillin and 100 µg/mL Streptomycin (Lonza, Verviers, Belgium).

Bevacizumab (Roche, Paris, France) was diluted with culture medium to working concentrations before use. SU1498 (EMD Chemicals, San Diego, USA), a selective VEGFR2 tyrosine kinase inhibitor [16], was prepared as a stock solution of 30 mM in DMSO, then diluted with culture medium to working concentrations before use. As a control to Bevacizumab, a stock solution containing the corresponding excipient was prepared with 60 mg/mL α,α trehalose dihydrate; 5.8 mg/mL sodium dihydrogen phosphate monohydrate and 1.5 mg di-sodium hydrogen phosphate dihydrate (all from Sigma Aldrich, Saint-Quentin Fallavier, France).

### 2. Assessment of VEGF secretion and VEGFR expression

VEGF secretion was assessed with the Quantikine ELISA kit for Human VEGF (R&D Systems, Abingdon, UK). U87 cells were seeded (3×10^5^ cells/well) in 24-well plates. After an overnight incubation, cells were incubated with or without SU1498 or Bevacizumab for 24 hours. The VEGF secretion was then assessed in the supernatant following the manufacturer's instructions.

VEGFR1 and VEGFR2 expressions were assessed at the protein level by FACS flow cytometry. Cells were harvested with 1 mM EDTA and adjusted in PBE buffer (PBS containing 0.5% BSA and 2 mM EDTA) to 4×10^6^ cells/mL. Phycoerythrin (PE)-conjugated anti VEGFR1 (clone 49560, R&D systems, Minneapolis, USA) and Alexa Fluor 647-conjugated anti VEGFR2 (clone HKDR-1, Biolegend, Saint Quentin Yvelines, France) were added for 30 min. PE-conjugated IgG1 (R&D systems, Minneapolis, USA) and Alexa Fluor 647-conjugated IgG1 (Biolegend, Saint-Quentin-en-Yvelines, France) were used as negative isotype controls. Cells were washed three times and resuspended in PBE buffer and 10,000 events were acquired on a BD LSR II flow cytometer. Results were analyzed using the Cyflogic sofware (Cyflo, Turku, Finland).

VEGFR1 and VEGFR2 expressions were assessed at the mRNA level by Quantitative Real-Time PCR, using hydroxymethylbilane synthase (HMBS) as housekeeping gene. Primer sequences were as follows: VEGFR1 (Flt-1, NM_002019) sense, 5′-AAGCAAACCACACTGGCTTC-3, antisense, 5′-CGGGGATTTCACTGTACATCT-3′; VEGFR2 (KDR, NM_002253) sense, 5′-TCTCTCTGCCTACCTCACCTG-3′, antisense, 5′-CGGCTCTTTCGCTTACTGTT-3′; HMBS (NM_000190) sense, 5′-ACCAAGGAGCTTGAACATGC-3′, antisense, 5′-GAAAGACAACAGCATCATGAG-3′. Cycle threshold values (crossing points, Cp) for each reaction were determined using LightCycler Software Version 4.0 (Roche Diagnostics, Meylan, France). PCR products were size fractionated on 1.5% agarose gels to confirm specific amplification of target and housekeeping genes.

### 3. Assessment of cell proliferation, cell cycle, apoptosis and cell morphology

For cell proliferation assay, U87 cells were seeded in 24-well plates (30,000 cells/well) and allowed to attach overnight. Cells were then treated for 24 or 72 h with different concentrations of Bevacizumab (from 10 ng/mL to 250 µg/mL) or SU1498 (from 1 µM to 30 µM) in triplicate wells. The cell viability was then assessed with the MTT assay following Mosmann, 1983 [22]. Briefly, the MTT reagent (thiazolyl blue tetrazolium bromide, Sigma Aldrich, Saint-Quentin Fallavier, France) was added to cells and optical density of the DMSO-dissolved formazan salts was measured after 3 hours incubation. The percentage of surviving cells is expressed as the ratio of optical density of treated cells versus untreated cells (control).

For bromodeoxyuridine (BrdU) incorporation assay, a colorimetric BrdU Cell Proliferation ELISA Kit (Abcam, Cambridge, UK) was used. U87 cells were either left untreated (control) or treated for 24 h with Bevacizumab (0.1 mg/mL) or SU1498 (10 µM), using quadruplicate wells per condition. BrdU was added 4 hours before the end of the incubation period. Cells were then fixed, DNA was denatured, and BrdU content was then assessed using a monoclonal anti-BrdU antibody following the manufacturer's instructions.

For cell cycle analysis, U87 cells were either left untreated (control) or treated for 24 h with Bevacizumab (0.1 mg/mL) or SU1498 (10 µM) in triplicate wells. At the end of the incubation period, cells were trypsinized and fixed in 75% ethanol for 30 min at 4°C. Cells were then resuspended in 200 µL PBS containing 50 µg/mL Propidium Iodide and 0.75 mg/mL RNase A. Cell cycle analysis was performed using a BD LSR II flow cytometer. 10,000 events per sample were acquired and results were analyzed using using Flowing Software 2.5.1 (Turku Centre for Biotechnology, University of Turku, Finland).

For apoptosis assay, U87 cells were either left untreated (control) or treated for 72 h with Bevacizumab (0.1 mg/mL) or SU1498 (10 µM) in triplicate wells. After trypsinization, apoptosis was assessed using the Annexin V–FITC Apoptosis Detection Kit (eBioscience, Paris, France) following the manufacturer's instructions. 5,000 events per sample were acquired on a BD LSR II flow cytometer. Results were analyzed using the Cyflogic sofware (Cyflo, Turku, Finland).

For morphological studies, U87 cells were seeded in 24-well plates (0.6×10^6^ cells/well), and treated for 48 hours with 0.1 mg/mL Bevacizumab or 10 µM SU1498. Cells were then fixed in 3.7% formaldehyde (5 min incubation at room temperature), permeabilized with 100% methanol (20 min incubation at room temperature), and stained with hematoxylin solution (Dako, Les Ulis, France).

For neutral lipid staining, U87 cells were seeded in 8-well Chamber Slides (Thermo Scientific, Rochester, NY, USA). Cells were either left untreated (control) or treated for 24 h with Bevacizumab (0.1 mg/mL), SU1498 (10 µM) or Linoleic Acid (200 µM) in duplicate wells. At the end of the incubation period, cells were fixed with 4% paraformaldehyde, stained with Oil Red ‘O’ Solution and counterstained with Hematoxylin Solution (both solutions from Millipore, Temecula, CA, USA) following the manufacturer's instructions.

### 4. ^1^H NMR spectroscopy

Subconfluent U87 cells (4×10^6^ cells/60 mm diameter cell culture dishes) were incubated for 24 hours and then treated or not with Bevacizumab (0.1 mg/mL); trehalose (0.24 mg/mL); or SU1498 (10 µM) for 24 hours. The cells (∼6×10^6^ cells/dish) were then harvested in 0.5 mL cold (4°C) PBS in deuterated water (Euriso-top, Gif-Sur-Yvette, France) using a cell scrapper and were washed twice. After centrifugation (3 min at 4°C, 300 g), 50 µL inserts were filled with 6 millions cells (using one insert per dish). Inserts were snap-frozen until NMR experiments. Ten inserts from different culture flasks were done for each condition (named technical replicates) according to standard procedures [19]. Cultures grown independently (in separate flasks and at different dates) were performed for each condition (named biological replicates). The metabolomic profiling of cells were investigated *in vitro* with high resolution-magic angle spinning proton magnetic resonance spectroscopy (HR-MAS). Spectra were acquired at 500 MHz on a Bruker NMR spectrometer (Bruker, Wissembourg, France), with a HRMAS probe. All experiments were performed at 294 K. Rotation rate was 4 kHz. Water signal was suppressed by a presaturation sequence using a low power irradiation at the water frequency. To remove broad signals produced by proteins and compounds exhibiting slow reorientation, a CPMG pulse sequence was used. The scan number was 128, the repetition rate of the spectra was 5 s. For resonance assignment purpose, TOCSY and JRES were also acquired. Spectra were first processed using NMRPipe [23] with an exponential function corresponding to 0.3 Hz line broadening prior to Fourier transforms. Spectra were phased and a baseline correction was applied between −0.5 and 10.5 ppm. The spectra were divided into 11,000 regions of 0.001 ppm width, called buckets. Each bucket was integrated and scaled using the probabilistic quotient normalization. For each condition, to limit variability due to technical steps, the mean of the 10 technical replicates has been done. Statistical analyses were performed on the mean spectra of the different biological replicates to evaluate metabolic impacts of trehalose, Bevacizumab and SU1498.

### 5. Statistical analyses and metabolites identification of HR MAS spectra

Principal Component Analysis (PCA) was conducted to detect any outliers based on NMR signal variability, defined as observations situated outside the 95% confidence region of the model. Orthogonal Projection to Latent Structure (OPLS) analysis was performed when no significant differences were seen with the PCA analysis. The ability of the model to describe data and to predict correctly new data is expressed by the value of the parameter R2 and Q2. R2 = 1 indicates a perfect description of the data while Q2 = 1 indicates a perfect prediction of new data.

Results are visualized by scores and loadings plots. The scores plot is showing the separation between groups. Scores are represented as a projection of the different samples on the predictive (Tpred) and the orthogonal (Torth) component. The loadings plot is showing the distribution of the corresponding variables responsible for the separation observed in the scores plot. PCA and OPLS were conducted using SIMCA-P12 (Umetrics) and in-house Matlab (Mathworks) code based on Trygg and Wold method [24].

For metabolites considered as discriminant by the multivariate analysis, an univariate analysis of variance (ANOVA) was performed to evaluate if the concentration of these metabolites were significantly affected by treatments. Concentrations of metabolites were calculated by integration of their NMR signal. To take into account the biological variations due to the fact that different cultures were grown independently, two factors were considered for the ANOVA: treatment (control, Bevacizumab, trehalose or SU1498) and culture (the date of the culture). Normal distribution was verified for all concentrations by performing a Lilliefors test. Pairwise comparisons were performed to determine which treatments were significantly different from the others.

## Results

### 1. U87 cells secrete VEGF and express VEGFR2

**Figure 1:**
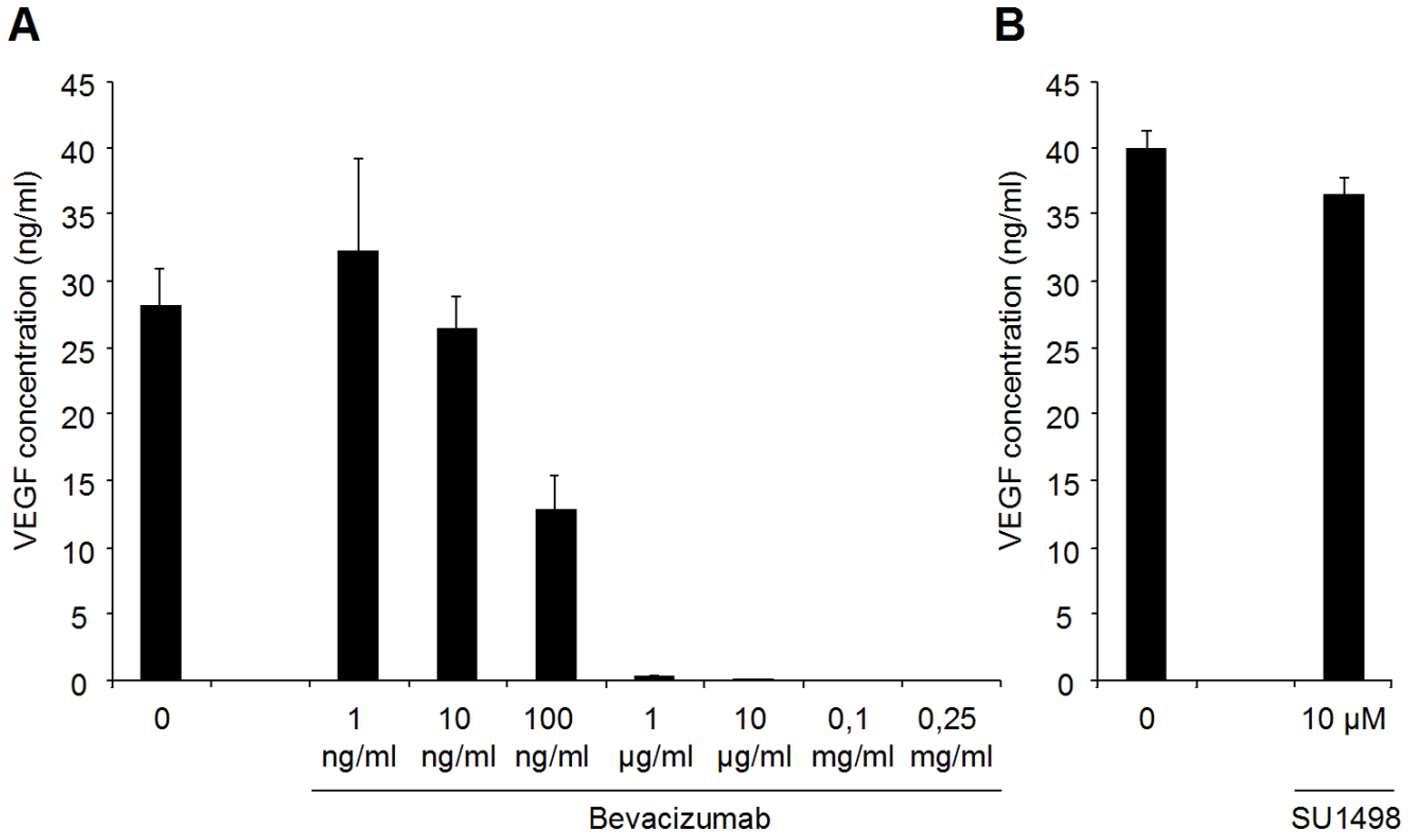
Effect of Bevacizumab and SU1498 on VEGF concentration in U87 cell culture supernatants. U87 cells were seeded (3×10^5^ cells/well) in 24-well plates. After an overnight incubation, cells were incubated with or without different concentrations of Bevacizumab (A) or 10 µM SU1498 (B) for 24 hours. The VEGF secretion was then assessed in the supernatants by ELISA. Data are expressed as ng/mL VEGF (mean ± standard deviation, n = 3 wells per condition). U87 cells release high levels of VEGF in the supernatant, which can be completely trapped by Bevacizumab concentrations above 1 µg/mL. On the contrary, treatment of U87 cells by 10 µM SU1498, a selective VEGFR2 inhibitor, has no impact on VEGF secretion.

**Figure 2:**
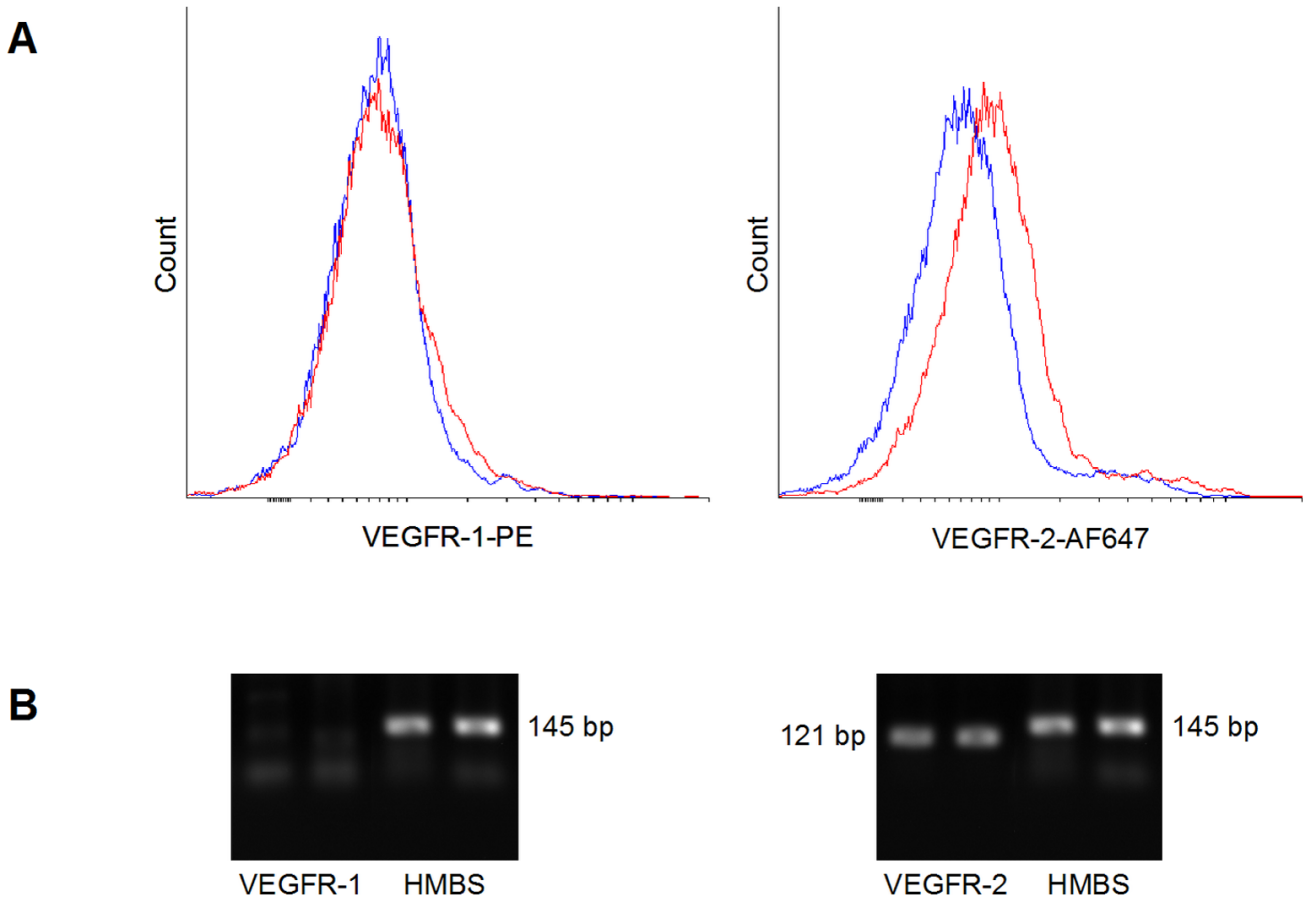
Expression of VEGFR1 and VEGFR2 by U87. (A) U87 cells were incubated with Phycoerythrin (PE)-conjugated anti VEGFR1 (left panel, red histogram) or with Alexa Fluor 647-conjugated anti VEGFR2 (right panel, red histogram). PE-conjugated IgG1 (left panel, blue histogram) and Alexa Fluor 647-conjugated IgG1 (right panel, blue histogram) were used as negative isotype controls. Representative histograms of duplicate experiments are shown. VEGFR1 and VEGFR2 expression was analysed by flow cytometry. U87 cells express low levels of VEGFR2, and no VEGFR1. (B) Total RNA was extracted from U87 cells and reverse transcribed. cDNA was quantified by quantitative real-time PCR (QRT-PCR). Expected sizes of PCR products were VEGFR1, 138 bp, VEGFR2, 121 bp and HMBS, 145 bp. The PCR products were size fractionated on 1.5% agarose gels and DNA was visualized by ethidium bromide staining. The results of two independent assessments of VEGFR1 and VEGFR2 are shown, using HMBS for normalization. Specific amplicons corresponding to VEGFR2, but not to VEGFR1, are detected.

In a first set of experiments, U87 cells were seeded (3×10^5^ cells/well) in 24-well plates and VEGF concentration in medium was assessed 24 hours later. U87 cells secrete high levels of VEGF (figure 1A). In addition, FACS analysis of U87 cells showed low expression levels of VEGFR2, and no VEGFR1 (figure 2A). These results were confirmed at the mRNA level by Quantitative Real-Time PCR (figure 2B). No expression of VEGFR1 could be detected. Crossing points (Cp) were 24.6±0.5 for VEGFR2 and 24.0±0.2 for HMBS, giving a VEGFR2/HMBS relative expression ratio of 0.68±0.17.

In a second set of experiments, we defined the optimal concentration of Bevacizumab needed to neutralize the secreted VEGF *in vitro*. U87 cells were seeded in 24-well plates. After a 24 h incubation, different concentrations of Bevacizumab (1 ng/mL to 0.25 mg/mL) were applied for 24 hours. Bevacizumab with concentration above 1 µg/mL completely trapped VEGF in the supernatant (figure 1A). Treatment of U87 cells by 10 µM SU1498, a selective VEGFR2 inhibitor, had no impact on VEGF secretion (figure 1B).

### 2. Impact of SU1498 and Bevacizumab on U87 proliferation

**Figure 3:**
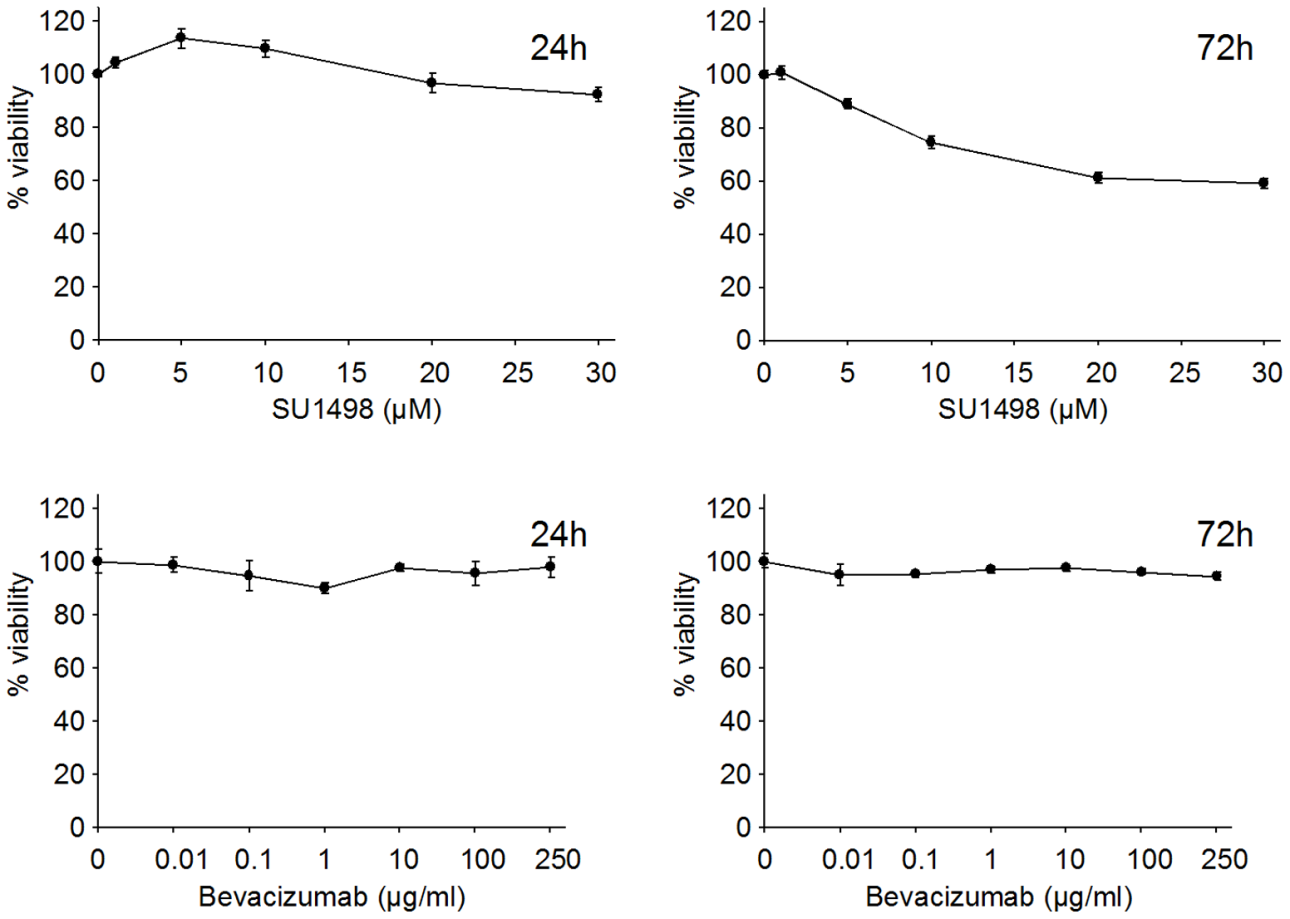
Effect of SU1498 and Bevacizumab on U87 cell proliferation. U87 cells were seeded in 24-well plates (30,000 cells/well) and were treated with different concentrations of SU1498 (from 1 µM to 30 µM) or Bevacizumab (from 10 ng/mL to 250 µg/mL). Viability was then assessed by a standard MTT assay. Bevacizumab has no significant impact on cell proliferation. SU1498 reduces cell proliferation with concentrations above 10 µM after a 72 h treatment, but no effect is seen at 24 hours.

The effect of Bevacizumab and SU1498 on cell proliferation was then assessed. U87 cells were treated for 24 h and 72 h with different concentrations of Bevacizumab (10 ng/mL to 1 mg/mL) and SU1498 (1 µM to 30 µM). Bevacizumab had no significant impact on cell proliferation as assessed by a MTT Assay. SU1498 reduced cell proliferation with concentrations above 10 µM after a 72 h treatment, but no effect was seen at 24 hours (figure 3). Concentrations of 0.1 mg/mL Bevacizumab and 10 µM SU1498 which are clinically relevant were selected for HRMAS experiments. Using these concentrations of Bevacizumab and SU1498, the doubling times of U87 cell population were 21.1, 21.5 and 26.3 hours respectively for control, Bevacizumab- and SU1498-treated cells as determined by growth curve analysis on a 72-hour incubation period (data not shown).

### 3. Impact of SU1498 and Bevacizumab on U87 cell cycle and apoptosis

**Figure 4:**
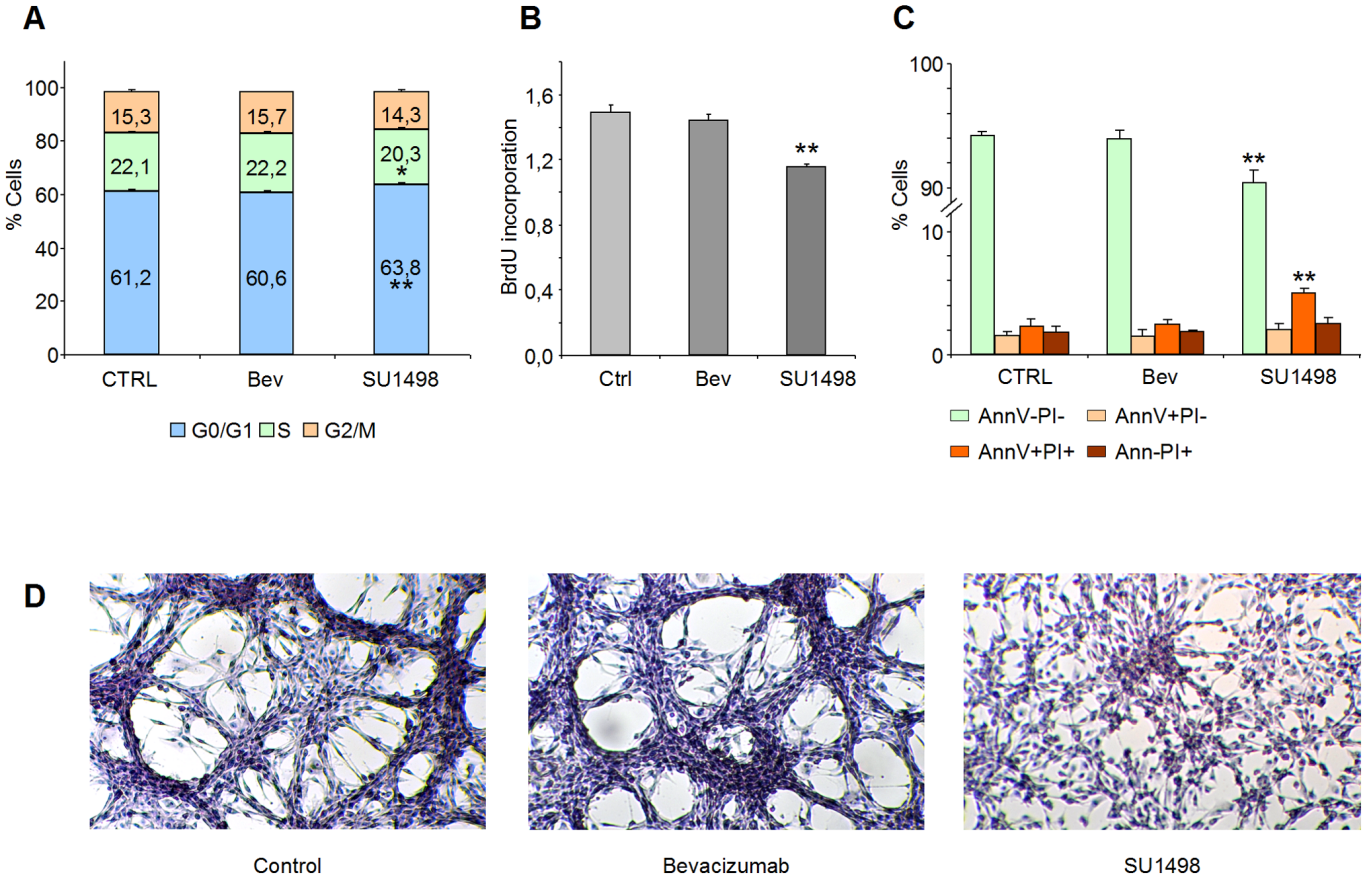
Impact of SU1498 and Bevacizumab on U87 cell cycle, apoptosis and morphology. (A) Impact of SU1498 and Bevacizumab on U87 cell cycle. U87 cells were treated for 24 h with Bevacizumab (0.1 mg/mL) or SU1498 (10 µM) in triplicate wells. After trypsinization, cells were fixed and stained with Propidium Iodide. Cell cycle analysis was performed using a flow cytometer. Bars represent mean + standard deviation (n = 3 wells per condition, *:p<0.05, **:p<0.01). SU1498 induces a slight increase in G0/G1 phase and a slight decrease in S phase when compared to untreated control (CTRL) cells. Bevacizumab treatment does not significantly impact cell cycle. (B) Impact of SU1498 and Bevacizumab on BrdU incorporation into U87 cells. U87 cells were treated for 24 h with Bevacizumab (0.1 mg/mL) or SU1498 (10 µM). BrdU incorporation into the newly synthesized DNA of replicating cells was assessed using a monoclonal anti-BrdU antibody and a subsequent colorimetric detection. Bars represent mean + standard deviation of optical density units proportional to BrdU content (n = 4 wells per condition, **:p<0.01). SU1498, but not Bevacizumab, induces a significant reduction in BrdU incorporation when compared to untreated (Ctrl) cells. (C) Impact of SU1498 and Bevacizumab on U87 cells apoptosis. U87 cells were treated for 72 h with Bevacizumab (0.1 mg/mL) or SU1498 (10 µM). Apoptosis was assessed using Annexin V–FITC/PI staining followed by flow cytometric detection. Bars represent mean + standard deviation (**:p<0.01). After 72 hours, SU1498 induces a slight but significant increase in the percentage of late apoptotic/necrotic Annexin V^+^/PI^+^, accompanied by a decrease in the percentage of viable (Annexin V^-^/PI^-^) cells, when compared to control (CTRL) cells. On the contrary, Bevacizumab treatment does not significantly impact the percentage of Annexin V^+^/PI^+^ cells. (D) Assessment of cell morphology. U87 cells were seeded in 24-well plates (0,6×10^6^ cells/well), treated for 48 hours with 0.1 mg/mL Bevacizumab or 10 µM SU1498, then stained with hematoxylin solution. Treatment with SU1498, but not Bevacizumab, induces a morphological modification, from a spindle-shaped to a fibroblast-like cell phenotype.

In order to provide further insight into the mechanism of action of SU1498 on cell growth inhibition, the effects of Bevacizumab and SU1498 on cell cycle and apoptosis of U87 cells were investigated. After a 24-hour incubation period, SU1498 induced a slight increase in G0/G1 phase (63.8±0.6%; p = 0.006) and a slight decrease in S phase (20.3±0.8, p = 0.02) when compared to control cells (G0/G1: 61.2±0.6%; S:22.2±0.1%). Bevacizumab treatment did not significantly impact cell cycle (G0/G1: 60.6±0.9%, p = 0.42; S:22.2±0.6%, p = 0.78) (figure 4A). Accordingly, SU1498, but not Bevacizumab, induced a significant reduction in BrdU incorporation (Ctrl: 1.49±0.05 OD unit; Bev: 1.45±0.03 OD unit, p = 0.19, SU1498: 1.16±0.02 OD unit, p = 0.00001) after a 24-hour incubation period (figure 4B).

After a 72-hour incubation, SU1498 induced a significant increase in the percentage of late apoptotic/necrotic Annexin V and PI double stained cells (4.95±0.42%, p = 0.003) when compared to control cells (2.35±0.56%). On the contrary, Bevacizumab treatment did not significantly impact the percentage of Annexin V^+^/PI^+^ cells (2.45±0.40%, p = 0.82) (figure 4C).

### 4. Impact of SU1498 and Bevacizumab on U87 morphology

The effect of a 48 h exposure to Bevacizumab and SU1498 on cell morphology was then assessed. The U87 cells were seeded in 24 well plates (0.6×10^6^ cells/well), treated with 0.1 mg/mL Bevacizumab or 10 µM SU1498 for 48 hours, then stained with hematoxylin solution. Treatment with SU1498, but not Bevacizumab, induced a morphological modification from a spindle-shaped to a fibroblast-like cell phenotype (figure 4D).

### 5. Bevacizumab fingerprint on U87 cells

**Figure 5:**
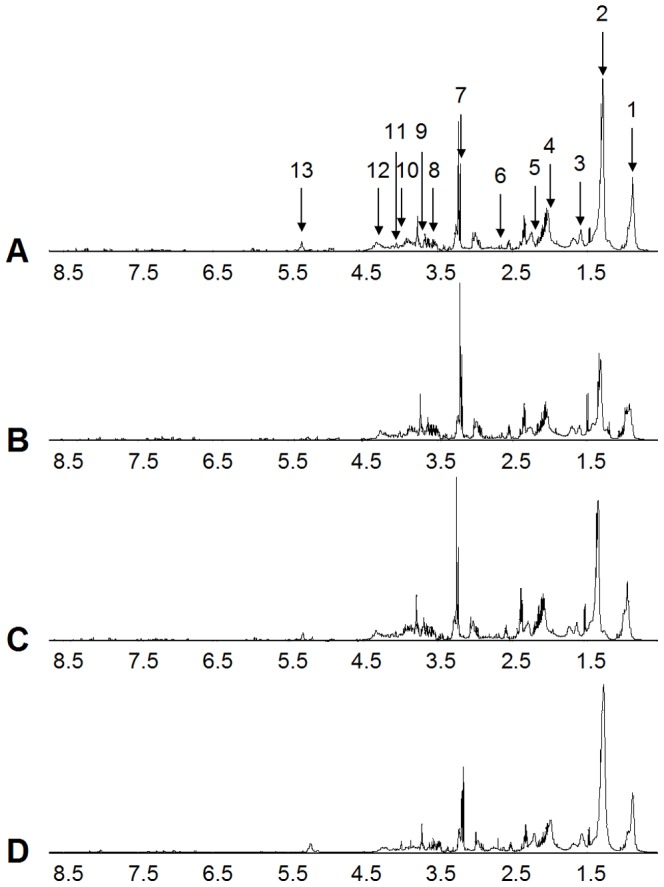
Mean Proton CPMG spectrum of control cells (A), cells incubated with trehalose (B), Bevacizumab (C) or SU1498 (D). The 1D spectra reveal the presence of principal lipid components [1: methyl; 2,3,4,5,6: methylen; 13: hydrogen of unsaturated hydrogencarbon from fatty acids of triglycerides or phospholipids] and certain metabolites [7,10,12: glycerophosphocholine; 8,9,11: myoinositol].

**Figure 6:**
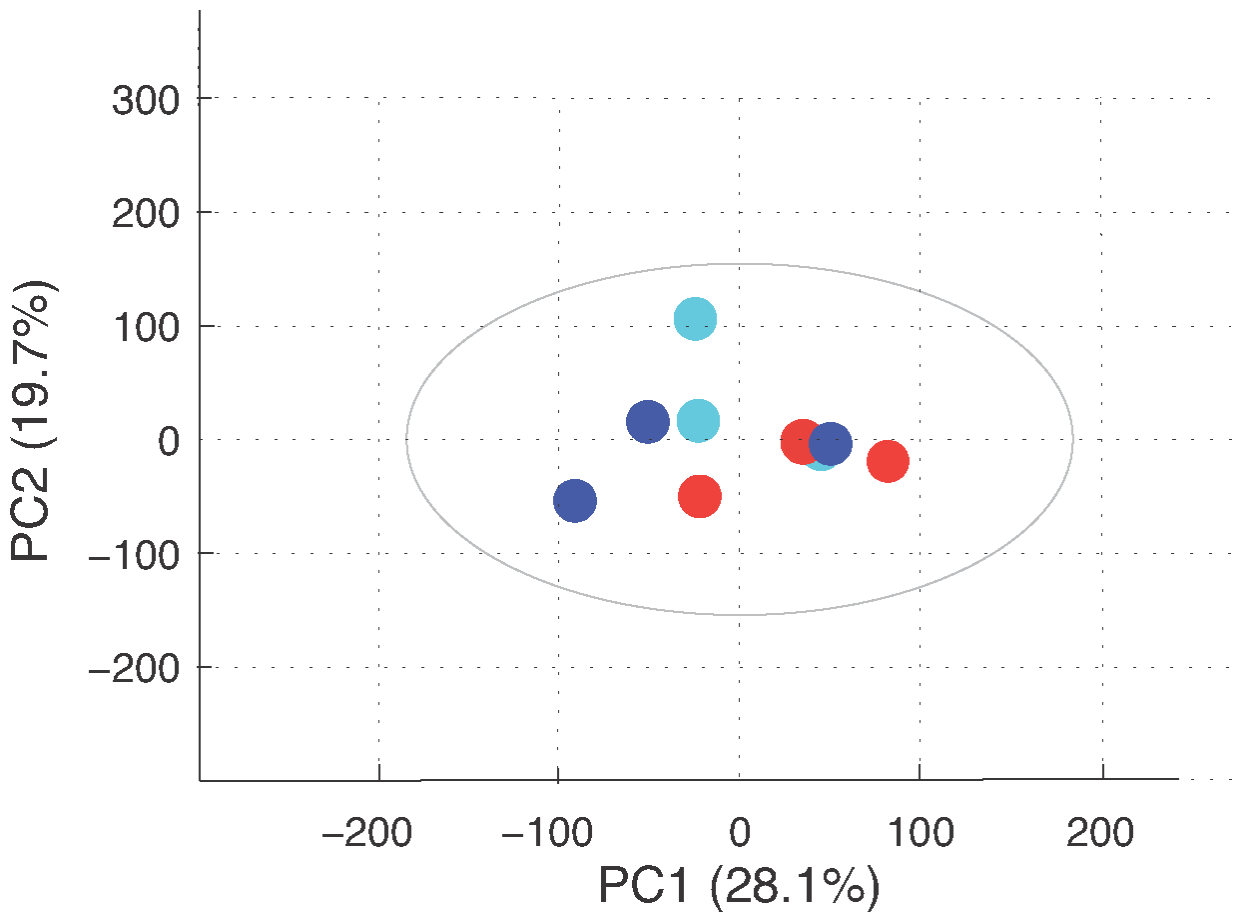
Score plot of two-component PCA model of proton NMR spectra for Bev-treated, trehalose-treated and ctrl cells. Each dot corresponds to a mean spectrum of 10 culture flasks: Light blue dots for control cells, red dots for trehalose-treated cells and dark blue dots for Bev-treated cells. The X and Y axis indicate the percentage of variance captured by each principal component. With this unsupervised PCA analysis, no group can be discriminated, showing that the maximum variability of the samples is not related to Bevacizumab treatment.

A HR MAS study was undergone to characterize the metabolic profiling of Bevacizumab treatment on U87 cells. The mean CPMG spectra, showing the main cell metabolites studied, are shown in figure 5. To study the Bevacizumab impact on U87 cells, a first statistical study was performed between control cells and Bev-treated cells, showing as difference the presence of the trehalose, which is an excipient contained in Bevacizumab. A second test set included 3 different culture sets (biological replicates) of trehalose-treated cells (each composed of 10 culture flasks), 3 different cultures of control cells (each composed of 10 culture flasks) and 3 different cultures of Bev-treated cells (each composed of 10 culture flasks). With the unsupervised PCA analysis, no group can be discriminated as illustrated by the scores plot realized on the biological replicates (figure 6), showing that the maximum variability of the samples was not related to Bevacizumab. No statistically validated OLPS model was obtained demonstrating that the metabolic impact of Bev is limited on U87 cells *in vitro*.

### 6. SU1498 fingerprint on U87 cells

**Figure 7:**
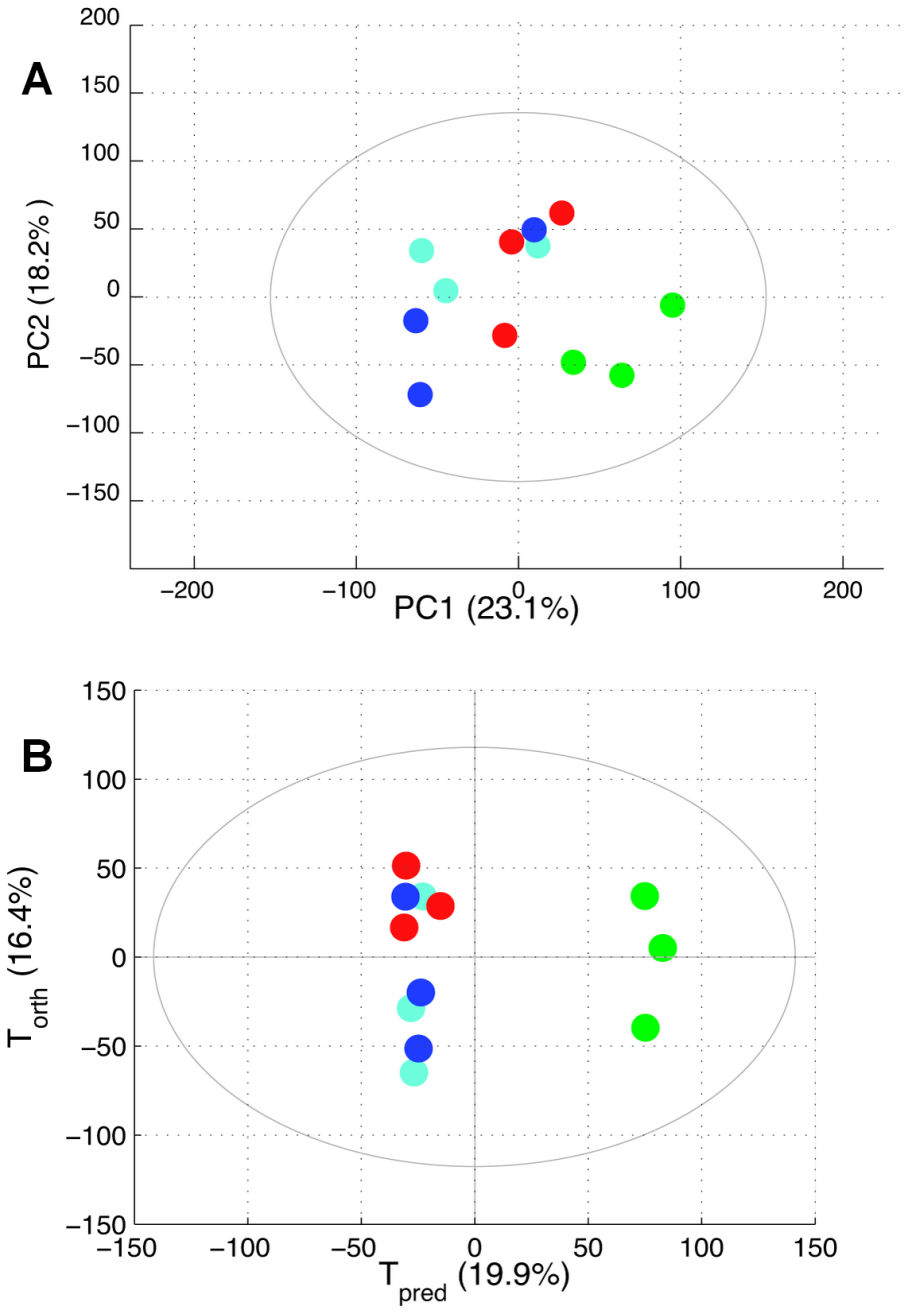
PCA score plot and OPLS score plot for SU1498-, Bev- or trehalose-treated and ctrl cells. On the PCA (A) and OPLS (B) score plots, each dot corresponds to a mean spectrum of 10 culture flasks: green dots for SU1498-treated cells; dark blue dots for Bev-treated cells, red dots for trehalose-treated cells and light blue dots for control cells. The X and Y axis indicates the percentage of variance captured by each principal component. A clear metabolic impact of SU1498 is observed whereas no metabolic impact can be seen for Bev.

**Figure 8:**
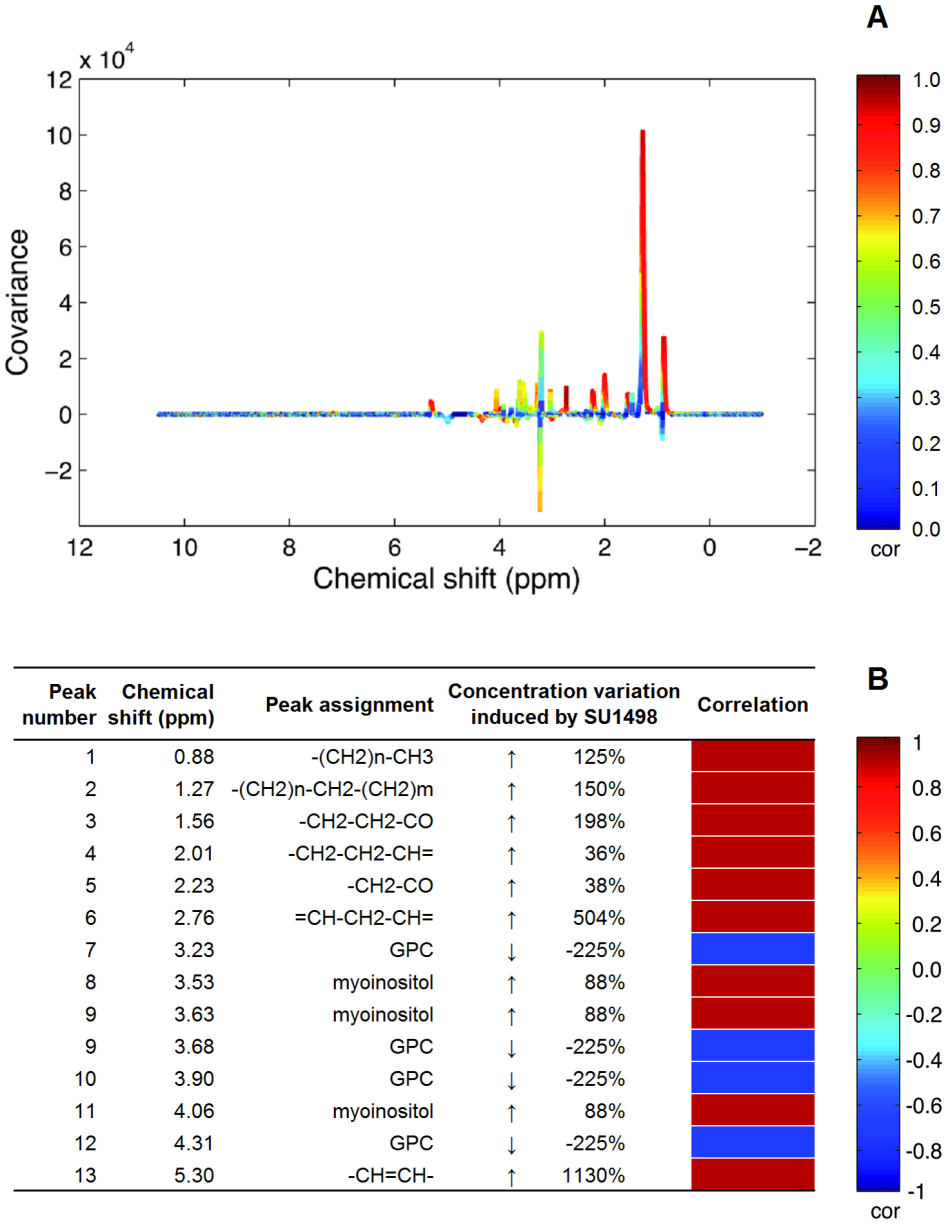
OPLS loading plot for SU1498-treated U87 cells (A), discriminant metabolites and heat-map of altered metabolites observed by ^1^H NMR spectroscopy (B). On the loading plot (A), positive signals correspond to increased metabolites in cells incubated with SU1498. The main metabolites modified by SU1498 treatment (B) are lipids (the CH = CH and CH = CHCH_2_CH = CH signals being the most impacted), and to a lesser extent, glycerophosphocholine and myoinositol.

As a second step, the effect of SU1498 was then studied. A principal component analysis (PCA) has been performed: 3 different biological replicates of U87 cells treated with 10 µM SU1498 (corresponding to 30 culture flasks), 3 different biological replicates of U87 cells treated with 0.1 mg/mL Bev (corresponding to 30 culture flasks); 3 different biological replicates of non-treated control cells (30 culture flasks) and 3 different sets of trehalose-treated cells (30 culture flasks). The corresponding score plot is presented in figure 7A. In this unsupervised analysis, a clear metabolic impact of SU1498 was observed. The maximum variability of these samples is mainly described by the differences observed between the samples treated with SU1498 and the others. In this PCA, the first axis represents the direction of maximum variation of the data, which corresponds to 23.1% of the whole variability. As shown on figure 7A, the first two directions are necessary to discriminate between SU1498 and other samples. Consequently, an OPLS model has been calculated to characterize the metabolomic effect of SU1498 on U87 cells. The score plot is presented in figure 7B. The predictive ability of the model (Q2Y) is 0.87. The area under the curve (AUC) is 1. The corresponding loadings coefficients plot is represented in figure 8A. Using 2D experiments and HMDB database, the main metabolites modified by SU1498 treatment were identified as lipids (the CH = CH and CH = CHCH_2_CH = CH signals being the most impacted, with increases of 1130% and 504% respectively), and to a lesser extent, glycerophosphocholine (−225%) and myoinositol (+88%) (figure 8B). Using the 120 samples, a 2-way ANOVA considering treatment and culture effects was performed on concentration of metabolites considered as discriminant in this OPLS model. For each peak of figure 8B, integration was realized to calculate the metabolite concentration. For each metabolite ANOVA result was used for pair wise comparison of the different treatment effects. We found that for all these metabolites, concentrations on SU1498 treated samples were significantly different from the other samples. According to this pair wise comparison no significant difference is observed between others treatments.

### 7. Impact of SU1498 and Bevacizumab on U87 lipid content

**Figure 9:**
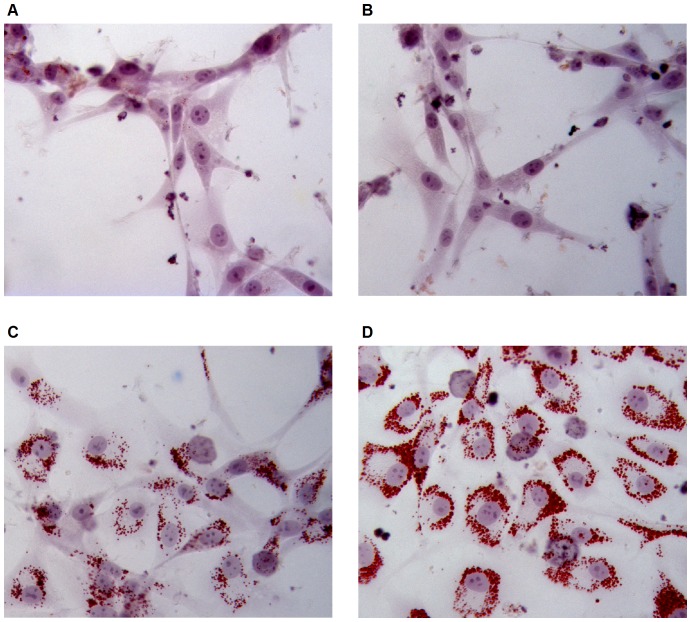
Impact of SU1498, Bevacizumab and Linoleic Acid on U87 cytoplasmic lipid content. U87 cells were seeded in 8-well chamber slides. Cells were left untreated (A) or were treated for 24 h with 0.1 mg/mL Bevacizumab (B), 10 µM SU1498 (C) or 200 µM Linoleic Acid (D) in duplicate wells. Cells were subsequently fixed with paraformaldehyde, stained with Oil Red ‘O’ solution and counterstained with Hematoxylin solution. Representative light microscope pictures (400x magnification) are shown. Treatment with SU1498, but not with Bevacizumab, induces the accumulation of lipid droplets in the cytoplasm of U87 cells. These droplets are similar to those seen when U87 cells are incubated with Linoleic Acid.

As HRMAS experiments demonstrated that treatment with SU1498 resulted in a significant alteration of lipid content in U87 cells, we investigated whether this could be visualized by intracellular lipid staining. U87 cells were treated with 0.1 mg/mL Bevacizumab, 10 µM SU1498 or 200 µM Linoleic Acid (positive control) for 24 hours, and then stained with Oil Red ‘O’ solution (figure 9). Treatment with SU1498 or Linoleic Acid, but not with Bevacizumab, induced the accumulation of lipid droplets in the cytoplasm of U87 cells.

## Discussion

Anti-angiogenic treatments targeting VEGF induce a high rate of radiological response in glioblastoma patients [8], [10], [11]. Several mechanisms can be advocated to explain these tumor responses: BBB normalization; glioma cell death resulting from reduced blood perfusion; or glioma cell death resulting from direct drug toxicity. BBB normalization is believed to be the main mechanisms underlying radiological response, but the degree to which the other mechanisms are involved remains unknown. This study was designed to assess the direct cytotoxicity of 2 anti-VEGF agents on tumor cells. This question is relevant as most glioma cells both secrete VEGF and express VEGFR, raising the question of an autocrine loop signalling [25]. The U87 cell line was selected because the expression of functional VEGFR2 has been reported by different teams [6], [16], [25], [26].

Using HRMAS, we here show that Bevacizumab does not significantly impact by itself the metabolism of U87 cells. This lack of impact on metabolism fits with the unmodified cell proliferation and morphology. Such an absence of effect of Bev on cells expressing VEGFR2 might seem surprising, but is in agreement with other studies, in which Bev did not significantly induce apoptosis or modify proliferation [26]–[28]. Only one study reported up-regulation of multiple genes under Bev, but at a very high concentration (5 mg/mL) that can hardly be reached in patients [6]. The emergence of an infiltrative phenotype under antiangiogenic treatments as been described in some animal models [6], [29]–[31], although the most recent studies suggest that it is not the case in patients [11], [32], [33]. The fact Bevacizumab did not affect glioma cells metabolism, might suggest that Bevacizumab is unlikely to modify glioma cells phenotype due to a therapeutic pressure on the VEGF autocrine loop. In agreement with that, we did not observe any phenotypic modification after a 3-month long term culture of U87 cells with 0.1 mg/mL Bevacizumab (data not shown).

Contrary to Bevacizumab, we showed that exposure to a VEGFR2-TKI (SU1498) induced a dramatic increase in lipids, and a decrease in glycerophosphocholine (GPC). Increase in lipids, and in particular in polyunsaturated fatty acids (PUFAs), is a well-documented sign of an apoptotic response with accumulation of lipid droplets prior to DNA fragmentation [34]–[36]. In a study on human breast cancer cells, increase in lipids resonating at 5.35, 1.3, and 0.9 ppm during cytotoxic drug treatment was associated with mitochondrial damage, lipid droplets development, and formation of autophagic vacuoles [37]. In glioma cells, it has been demonstrated that PUFAs accumulate into BT4C glioma during cisplatin or gene therapy-induced programmed cell death (PCD), with pattern recognition identifying CH = CH and CH = CHCH_2_CH = CH as the most significant in monitoring the dynamics of PCD [34], [38]. In the present work, and in line with these findings, we identified CH = CH and CH = CHCH_2_CH = CH patterns as the most impacted by exposure to SU1498, with increases of 1130% and 504% respectively. Interestingly, HRMAS experiments were performed after 24 hours exposure to SU1498, i.e. at a time point when no toxicity was evidenced by the MTT reduction assay, emphasizing the sensitivity of the HRMAS to detect early drug-induced alterations of cancer cells. Treatment of U87 cells with SU1498 resulted in the accumulation of cytoplasmic lipid droplets after 24 hours and in an increase of apoptotic cell population after 72 hours. Interestingly, exogenously added PUFAs (Oleic Acid, Linoleic Acid or α-Linolenic Acid) resulted in a similar accumulation of cytoplasmic lipid droplets after 24 hours in U87 cells, without any impact on cell proliferation after 72 hours (data not shown), indicating that PUFAs accumulation is not responsible for cell apoptosis in our model but rather constitutes an early marker of apoptosis. In addition to apoptosis rate enhancement, a slight impact of SU1498 on cell cycle progression (S-phase entry) was observed in the present work. This is in agreement with a previous study [25] showing that blockade of VEGFR2 by SU1498 abrogated the VEGF-mediated enhancement of glioma cell growth and viability. In another study [39], Cediranib, a potent inhibitor of VEGFRs, which also targets KIT and PDGFRA, was shown to induce a significant reduction of U251 glioma cells in S phase and a higher percentage of apoptotic cells. VEGFR2 inhibition by SU1498 or Cediranib in glioma cells, including U87, suppressed different intracellular signaling cascades, such as the focal adhesion kinase (FAK), the mitogen-activated protein kinase ERK1/2, or the phosphatidylinositol 3 kinase (PI3K)/AKT pathways [16], [25], [39]. In the present work, changes in myoinositol and GPC induced by SU1498 treatments were less prominent than changes in PUFAs. Again, this is in line with the findings of Griffin et al [35], who noticed relatively few changes for choline-containing metabolites in BT4C rat glioma following the induction of programmed cell death. These metabolites were uncorrelated with temporal progression through PCD [40].

The discrepancy in the effects of Bev and SU1498 was unexpected for two drugs targeting the same VEGF pathway. Although we acknowledge that only one cell line was studied here, a similar discrepancy was reported in another glioma cell line [16]. One possibility is that SU1498 might be not fully selective for VEGFR2. However, even if other targets are partially inhibited with SU1498, the fact that Bev poorly impacts the cell metabolism remains relevant. This observation might reflect incomplete neutralization by Bevacizumab, at the glycocalyx level, of the continuously secreted VEGF. VEGF binds to heparan sulfate and is retained on the cell surface and in the extracellular matrix [41], [42]. It has been shown that large macromolecules, such as 70-kDa dextran, do not penetrate the intact glycocalyx [43]. The transport of Bevacizumab (approximate molecular weight of 149 kDa) across the glycocalyx layer might similarly be hindered, thereby allowing for persistent VEGF/VEGFR2 signalling. Alternatively, as it was shown for melanoma cells [44], an intracrine VEGF/VEGFR2 signalling that allows the cells to stimulate their own survival pathways without the need for exogenous secreted VEGF might be present in U87 cells. Such an intracrine loop would be protected from antibody blockade but accessible to TKI, therefore explaining the differential effects of Bevacizumab and SU1498.

In patients, magnetic resonance spectroscopy (MRS) is used to study the tumor metabolism *in vivo*, and might thus help to differentiate tumor cytotoxivity from vascular effects. Choline-containing compounds are typically elevated in glioblastomas, while N-acetylaspartate (NAA), regarded as a neuronal marker, is decreased [15]. Interestingly, a decrease in the Choline/Creatine and an increase in the NAA/Choline ratios have been observed in patients treated with Bev [45], [46]. As Bev has no apparent impact on tumor cells *in vitro*, and does not modify overall survival in patients [11], these ratios are thus not reliable to assess direct drug cytotoxicity. The dramatic increase in lipids observed in our *in vitro* study with SU1498, suggests that lipids might be a more relevant surrogate marker to assess the toxicity of a given drug in patients. Interestingly, lipids tend to increase under Cediranib, a TKI targeting VEGFR, in a series of patients undergoing MRS [47]. Further clinical studies with MRS are needed to assess the validity of the lipid signal as an early marker of tumor cell death in treated patients.
